# *Ecklonia cava* Extract and Dieckol Attenuate Cellular Lipid Peroxidation in Keratinocytes Exposed to PM10

**DOI:** 10.1155/2018/8248323

**Published:** 2018-03-06

**Authors:** Jeong-won Lee, Jin Kyung Seok, Yong Chool Boo

**Affiliations:** ^1^Department of Molecular Medicine, Cell and Matrix Research Institute, BK21 Plus KNU Biomedical Convergence Program, School of Medicine, Kyungpook National University, 680 Gukchaebosang-ro, Jung-gu, Daegu 41944, Republic of Korea; ^2^Ruby Crown Co., Ltd., 201 Kyungpook National University Business Incubation Center, 80 Daehak-ro, Buk-gu, Daegu 41566, Republic of Korea

## Abstract

Airborne particulate matter can cause oxidative stress, inflammation, and premature skin aging. Marine plants such as* Ecklonia cava* Kjellman contain high amounts of polyphenolic antioxidants. The purpose of this study was to examine the antioxidative effects of* E. cava* extract in cultured keratinocytes exposed to airborne particulate matter with a diameter of <10 *μ*m (PM10). After the exposure of cultured HaCaT keratinocytes to PM10 in the absence and presence of* E. cava* extract and its constituents, cell viability and cellular lipid peroxidation were assessed. The effects of eckol and dieckol on cellular lipid peroxidation and cytokine expression were examined in human epidermal keratinocytes exposed to PM10. The total phenolic content of* E. cava* extract was the highest among the 50 marine plant extracts examined. The exposure of HaCaT cells to PM10 decreased cell viability and increased lipid peroxidation. The PM10-induced cellular lipid peroxidation was attenuated by* E. cava* extract and its ethyl acetate fraction. Dieckol more effectively attenuated cellular lipid peroxidation than eckol in both HaCaT cells and human epidermal keratinocytes. Dieckol and eckol attenuated the expression of inflammatory cytokines such as tumor necrosis factor- (TNF-) *α*, interleukin- (IL-) 1*β*, IL-6, and IL-8 in human epidermal keratinocytes stimulated with PM10. This study suggested that the polyphenolic constituents of* E. cava*, such as dieckol, attenuated the oxidative and inflammatory reactions in skin cells exposed to airborne particulate matter.

## 1. Introduction

Air pollution of natural and artificial origins presents a significant concern. Particulate matter of less than 10 micrometers (PM10) suspended in the atmosphere has become a major threat to human health [1, 2]. Because PM10 is too small to be caught in the nasal cavity and bronchial cilia, it can deeply penetrate the lungs and cause inflammation, asthma, chronic bronchitis, and airway obstruction [3, 4]. PM10 can infiltrate blood vessels and then circulate throughout the body, which may cause cardiovascular and cerebrovascular diseases [5]. The particles can also attack eyes, causing various diseases such as allergic conjunctivitis and dry eye syndrome [6]. PM10 penetrates the skin through pores and other inflamed sites, aggravating skin diseases such as atopic dermatitis, acne, and psoriasis [7]. Furthermore, airborne particles are associated with premature skin aging [8].

The best strategy for the prevention and alleviation of airborne particle-induced diseases may be to reduce air pollution and the chance of exposure to air pollution. It is recommended that people minimize outdoor activity and wear a hat, mask, glasses, and long sleeves to protect the body from airborne particles. Although the skin is considered to be a physical barrier to the outer environment, the size and reactivity of PM10 are such that it can clog skin pores and induce inflammation. Therefore, dermatological and cosmetic approaches are needed to attenuate the oxidative and inflammatory reactions that arise from PM10 exposure.

Natural polyphenolic compounds may mitigate oxidative stress and inflammation as a result of exposure to PM10. In a previous study, chocolate reduced cardiac inflammation in mice exposed to urban air pollution [9]. The extract of* Eucheuma cottonii* attenuated inflammation and oxidative stress induced by chronic exposure to coal dust in rats [10]. Pomegranate peel extract and punicalagin attenuated the PM10-induced adhesion of THP-1 cells to endothelial cells, which was indicative of its anti-inflammatory activity against particulate matter [11].

Marine plants have emerged as a potential resource of bioactive compounds for the development of cosmeceutical ingredients [12]. Marine algae such as* Ecklonia cava *Kjellman have been shown to inhibit cellular melanin synthesis in murine melanoma B16/F10 cells [13, 14]. In our previous study, the extract of* Phyllospadix iwatensis* Makino was shown to inhibit human tyrosinase activity and melanin synthesis in human dermal melanocytes [15]. However, little is known about the antioxidant effects of marine plant extracts in skin cells that have been exposed to air pollution. In our preliminary experiments, 50 marine plants indigenous to Korea were analyzed to quantify the total phenol content (TPC). Among these plants, the* E. cava* extract was found to have the highest TPC. Therefore, we examined the antioxidant effects of* E. cava* extract in keratinocytes exposed to PM10.

## 2. Materials and Methods

### 2.1. Reagents

A standardized fine dust (PM_10_-like) (European Reference Material ERM-CZ120) and gallic acid (purity > 98%) were purchased from Sigma-Aldrich (St. Louis, MO, USA). Eckol (purity > 98%) and dieckol (purity > 98%) were purchased from National Development Institute of Korean Medicine (Gyeongsangbuk-do, Republic of Korea).

### 2.2. Plant Extracts

The extracts of 50 different marine plants, extracted with 80% (v/v) aqueous ethanol, were purchased from Jeju Biodiversity Research Institute of Jeju Technopark (Jeju, Korea). The list of the plant extracts has been previously reported [15].

### 2.3. Determination of TPC

The TPC of each plant extract was determined using the Folin-Ciocalteau method. The extract was dissolved in 95% ethanol at 1.0 mg mL^−1^, and 20 *μ*L aliquots were added to a 96-well microplate. A calibration curve using gallic acid (100–500 *μ*g mL^−1^) was established. Folin-Ciocalteau reagent (Sigma-Aldrich) was diluted 10-fold in water and 100 *μ*L was added to each well, which was reacted at 18–21°C (room temperature) for 5 min. Subsequently, 80 *μ*L of 7.5% Na_2_CO_3_ solution was added to the mixture and then allowed to stand for 60 min in the dark. The absorbance of the mixture was measured at 765 nm using a SPECTROstar Nano microplate reader (BMG LABTECH GmbH, Ortenberg, Germany) and the calculated TPC value was expressed as *μ*g gallic acid equivalent (GAE) per mg extract (*μ*g GAE mg^−1^ extract).

### 2.4. Preparation of* E. cava* Extracts and Fractions

The extract of* Ecklonia cava* Kjellman was prepared in our laboratory from the plant materials purchased from Jayeoncho (http://www.jherb.com) (Seoul, Korea). The dried* E. cava* (500 g) was extracted with 80% aqueous ethanol (2.5 L) for 7 days at room temperature. The extract solution was evaporated under reduced pressure to obtain the crude extract (71.5 g). The extract (4.2 g) was suspended in water (WT) (50 mL) and partitioned sequentially with an equal volume of methylene chloride (MC), ethyl acetate (EA), and* n*-butyl alcohol (BA). These fractions were evaporated under reduced pressure to yield four fractions: the MC (0.38 g), EA (0.72 g), BA (0.88 g), and WT fractions (2.2 g).

### 2.5. High-Performance Liquid Chromatography (HPLC)

HPLC was performed by using a Gilson HPLC system (Gilson, Inc., Middleton, WI, USA) with a 321 pump and UV/VIS 151 detector. The separation was performed on a 5 *μ*m Hector-M C_18_ column (4.6 mm × 250 mm) (RS Tech Co., Daejeon, Korea) using a mobile phase of 0.1% phosphoric acid (A) and acetonitrile (B). The gradient program was set as follows: 0–30 min, a linear gradient from 0 to 100% B; 30–40 min, 100% B. The flow rate was 0.6 mL min^−1^ and the detector was set at 280 nm.

### 2.6. Cell Culture

The cells were cultured in a closed incubator at 37°C in humidified air containing 5% CO_2_. HaCaT cells (an immortalized human keratinocyte cell line) were grown in DMEM/F‐12 medium (GIBCO‐BRL, Grand Island, NY, USA) supplemented with 10% fetal bovine serum, antibiotics (100 U mL^−1^ penicillin, 100 *μ*g mL^−1^ streptomycin, and 0.25 *μ*g mL^−1^ amphotericin B), and 10 *μ*g mL^−1^ hydrocortisone. Primary human epidermal keratinocytes from adult human donors (Invitrogen, Waltham, MA, USA) were cultured in EpiLife medium (Gibco BRL) supplemented with 10% EpiLife defined growth supplement (#S0125) and antibiotics.

### 2.7. Treatments with PM10

The cells were plated onto 6-well culture plates (SPL Life Sciences, Pocheon, Korea) at 8 × 10^4^ cells/well, cultured for 24 h, and then treated with PM10 at various concentrations (3–100 *μ*g mL^−1^) for 48 h. In some experiments, the cells were treated with a fixed concentration of PM10 (100 *μ*g mL^−1^) in the absence and presence of varied concentrations of a test material.

### 2.8. Cell Viability Assay

The cells were plated on 48-well culture plates at 1.6 × 10^4^ cells/well and the cell viability was assessed by an MTT assay, which assesses the cellular metabolic activity of the reduction of 3‐[4,5‐dimethylthiazol‐2‐yl]‐2,5‐diphenyl tetrazolium bromide (MTT) to formazan dye. The cells were washed with phosphate-buffered saline (PBS) and incubated in culture medium (200 *μ*L) containing 1 mg mL^−1^ MTT (Amresco, Solon, OH, USA) for 2 h at room temperature. After the medium was removed, the formazan dye in the cells was solubilized in dimethyl sulfoxide (200 *μ*L). The resulting dye solution was placed in a 96-well plate and the absorbance was determined at 595 nm by using a SPECTROstar Nano microplate reader.

### 2.9. Assay of Lipid Peroxidation

The cells were plated in 100 mm culture dishes at 5 × 10^5^ cells/dish and the cellular lipid peroxidation was assessed through the measurement of 2-thiobarbituric acid-reactive substances (TBARS) [16]. The cell extracts were prepared by using cell lysis buffer (20 mM Tris-Cl, 2.5 mM EDTA, 1.0% SDS, pH 7.5). The assay mixture consisted of cell extract (300 mg protein), 100 *μ*L lysis buffer, 50 *μ*L* m*-phosphoric acid, and 350 *μ*L 0.9% 2-thiobarbituric acid (Sigma-Aldrich), which was then heated in a boiling water bath for 45 min. After cooling, 500 *μ*L BA was added to the mixture, which was then vortex-mixed and centrifuged at 10,000 ×g for 15 min to produce two separate layers. The fluorescence intensity of the BA layer (excitation at 540 nm and emission at 590 nm) was measured by using a Gemini EM fluorescence microplate reader (Molecular Devices).

### 2.10. Quantitative Reverse-Transcriptase Polymerase Chain Reaction (qRT-PCR) Analysis

The mRNA levels of TNF-*α*, IL-1*β*, IL-6, and IL-8 were determined by qRT-PCR. Cellular RNA was extracted with an RNeasy kit (Qiagen, Valencia, CA, USA) and was used for the preparation of cDNA by using a High Capacity cDNA Archive Kit (Applied Biosystems, Foster City, CA, USA). Gene-specific primers were purchased from Macrogen (Seoul, Korea). The sequences of the primers were as follows: TNF-*α* (TNF, NM_000594.3), 5′-TGCTCCTCACCCACACCAT-3′ (forward) and 5′-GAGATAGTCGGGCCGATTGA-3′ (reverse); IL-1*β* (IL1B, NM_000576.2), 5′-CCTGTCCTGCGTGTTGAAAGA-3′ (forward) and 5′-TGTCCTGCAGCCACTGGTTC-3′ (reverse); IL-6 (IL6, NM_001318095.1, NM_000600.4), 5′-AAGCCAGAGCTGTGCAGATGAGTA-3′ (forward) and 5′-TGTCCTGCAGCCACTGGTTC-3′ (reverse); IL-8 (IL8, NM_000584.3), 5′-CTGCGCCAACACAGAAATTA-3′ (forward) and 5′-ACTTCTCCACAACCCTCTGC-3′ (reverse); glyceraldehyde 3-phosphate dehydrogenase (GAPDH, NM_002046.3), 5′-ATGGGGAAGGTGAAGGTCG-3′ (forward) and 5′-GGGGTCATTGATGGCAACAA-3′ (reverse). The qRT-PCR was conducted with a StepOnePlus™ Real-Time PCR System (Applied Biosystems). The reaction mixture (20 *μ*L) consisted of SYBR® Green PCR Master Mix (Applied Biosystems), cDNA (60 ng), and gene-specific primer sets (2 pmole). The thermal cycling parameters to be used for the PCR reactions were 50°C for 2 min, 95°C for 10 min, 40 amplification cycles of 95°C for 15 s and 60°C for 1 min, and a dissociation step. The mRNA levels of each gene were calculated relative to that of GAPDH by using the comparative threshold cycle method.

### 2.11. Enzyme-Linked Immunosorbent Assay (ELISA)

The protein levels of TNF-*α*, IL-1*β*, IL-6, and IL-8 in the culture medium were determined using respective ELISA kits (K0331131, K0331800, K0331194, and K0331216; Koma Biotech, Seoul, Korea). Briefly, cell culture media or standard protein solutions (100 *μ*L) were transferred to microplate wells containing immobilized antibodies for each protein, followed by the incubation for 2 h at room temperature. The wells were then washed and incubated for 2 h with biotinylated antibodies for each protein and incubated for 45 min with horseradish peroxidase-conjugated streptavidin. The wells were washed and 3,3′,5,5′-tetramethylbenzidine substrate solution was added to initiate enzymatic color development. After 30 min, a stop solution was added to terminate the reaction and the absorbance was measured at 450 nm. The concentrations of cytokines were estimated from each standard curve.

### 2.12. Statistical Analysis

The data were presented as mean ± standard errors (SE) of three or more independent experiments. The significance of the differences between groups was determined by using Student's *t*-test and value of *p* < 0.05 was considered statistically significant.

## 3. Results

**Figure 1:**
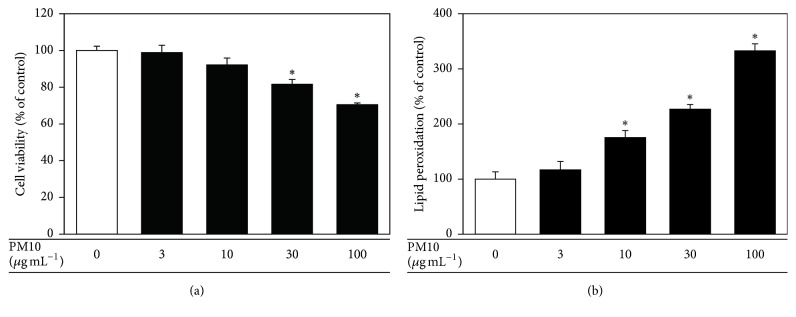
The effects of PM10 on viability of and lipid peroxidation in cultured HaCaT keratinocytes. The cells were treated with PM10 at the indicated concentrations for 48 h, followed by the determination of the cell viability (a) and cellular lipid peroxidation (b). The data are presented as a percentage of the control values (mean ± SE, *n* = 3). ^*∗*^*p* < 0.05 versus control.

When human HaCaT keratinocytes were treated with PM10 (3 to 100 *μ*g mL^−1^ for 48 h), their viability decreased in a dose-dependent manner. In addition, PM10 increased lipid peroxidation, as determined by the levels of TBARS (Figure 1(b)).

Of the 50 plant extracts, the extract of* Ecklonia cava* Kjellman had the highest TPC (89 *μ*g GAE mg^−1^ extract), followed by* Distromium decumbens* (Okamura) Levring,* Acrosorium yendoi *Yamada*, Eisenia bicyclis* (Kjellman) Setchell, and* Martensia denticulata* Harvey, as shown in Table 1. Thus, further studies focused on* E. cava *extract.

**Figure 2:**
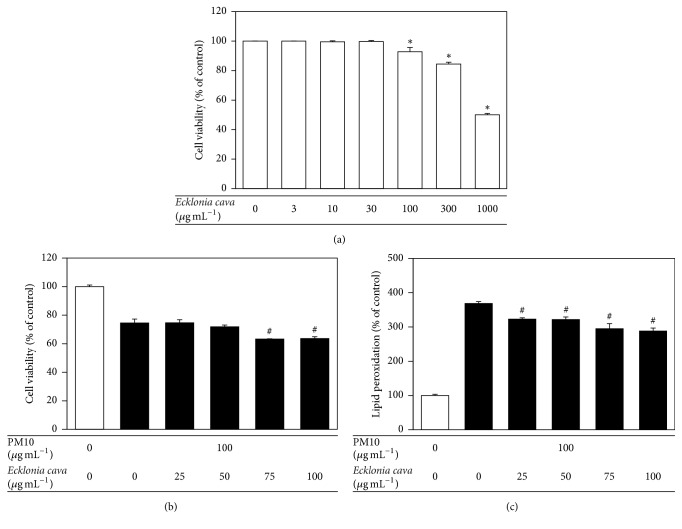
The effects of* E. cava* extract on viability of and lipid peroxidation in HaCaT keratinocytes exposed to PM10. In (a), the cells were treated with* E. cava* extract at the indicated concentrations for 48 h. In (b) and (c), the cells were exposed to PM10 (100 *μ*g mL^−1^) for 48 h in the absence and presence of* E. cava* extract at the indicated concentrations. The cell viability (a, b) and cellular lipid peroxidation (c) were determined. The data are presented as the percentage of the control value (mean ± SE, *n* = 3). ^*∗*^*p* < 0.05 versus control and ^#^*p* < 0.05 versus PM10 control.

*E. cava* extract was cytotoxic to HaCaT cells at concentrations above 100 *μ*g mL^−1^ (Figure 2(a)). In the following experiments, the cells were treated with* E. cava* extract at 25, 50, 75, or 100 *μ*g mL^−1^ and then exposed to 100 *μ*g mL^−1^ PM10 for 48 h. At 25~50 *μ*g mL^−1^*, E. cava* extract had no significant effect on the viability of HaCaT cells exposed to PM10 (Figure 2(b)), but significantly attenuated cellular lipid peroxidation (Figure 2(c)). At higher concentrations*, E. cava* extract attenuated cellular lipid peroxidation more effectively (Figure 2(c)) but it also decreased the cell viability (Figure 2(b)).

**Figure 3:**
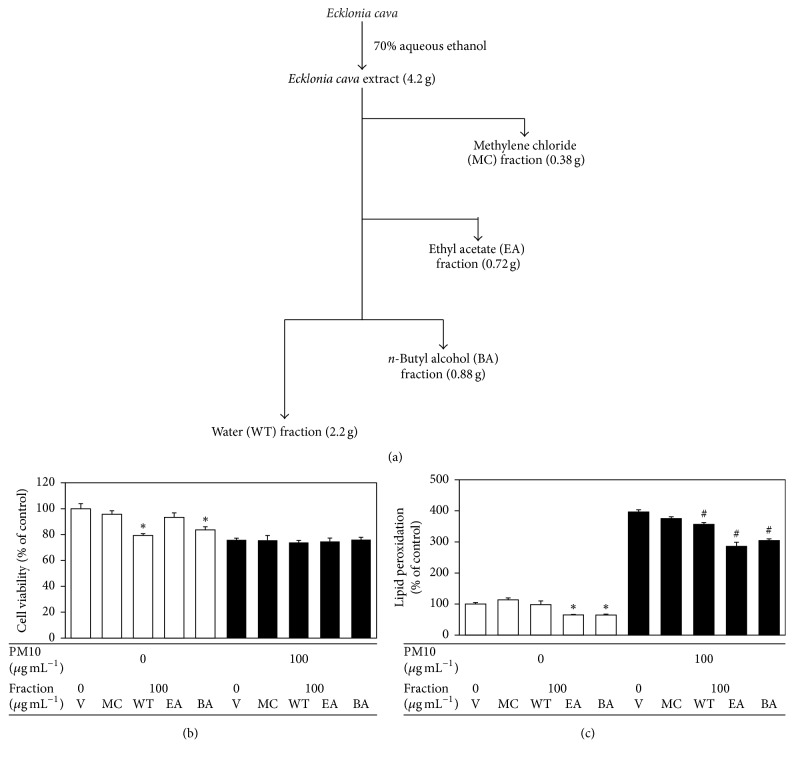
The effects of various solvent fractions of* E. cava* extract on viability of and lipid peroxidation in HaCaT keratinocytes exposed to PM10. In (a),* E. cava* extract was fractionated into the methylene chloride (MC), ethyl acetate (EA),* n*-butyl alcohol (BA), and water (WT) fractions. In (b) and (c), the cells were exposed to PM10 (100 *μ*g mL^−1^) for 48 h in the absence and presence of each fraction (100 *μ*g mL^−1^). The cell viability (b) and cellular lipid peroxidation (c) were determined. The data are presented as the percentage of the control values (mean ± SE, *n* = 3). ^*∗*^*p* < 0.05 versus control and ^#^*p* < 0.05 versus PM10 control.

Cells were treated with various solvent fractions of* E. cava* extract at 100 *μ*g mL^−1^ and then exposed to 100 *μ*g mL^−1^ PM10 for 48 h. In the absence of PM10, the water and* n*-butyl alcohol fraction of* E. cava* extract showed significant cytotoxicities, whereas the ethyl acetate fraction had no effect on the cell viability in comparison with the control (Figure 3(b)). All solvent fractions had no effects on the viabilities of the cells exposed to PM10 (Figure 3(b)). In the absence of PM10, the ethyl acetate and* n*-butyl alcohol fraction decreased the basal levels of lipid peroxidation (Figure 3(c)). The ethyl acetate fraction attenuated the PM10-stimulated cellular lipid peroxidation more effectively compared with other solvent fractions (Figure 3(c)).

**Figure 4:**
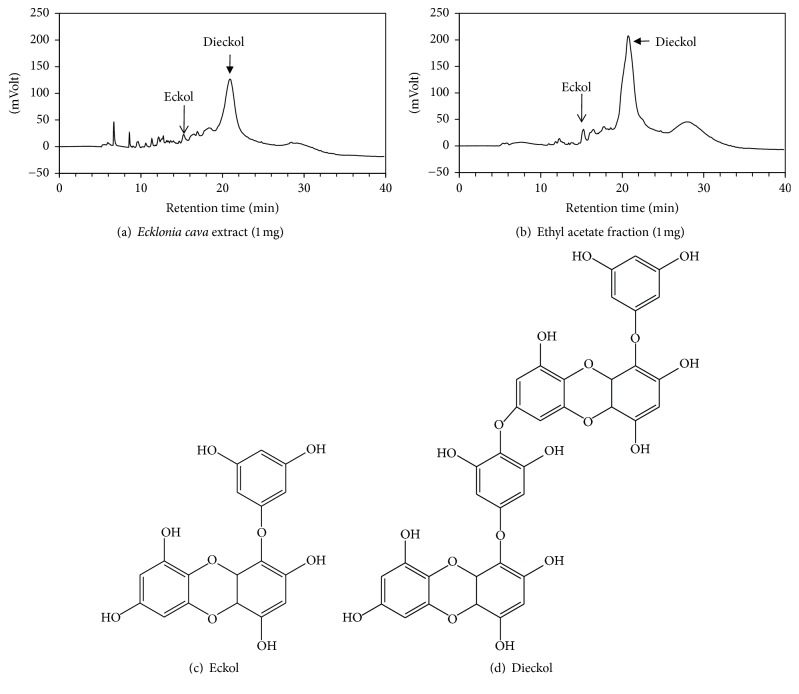
HPLC of* Ecklonia cava* extract and the chemical structures of eckol and dieckol. Typical HPLC patterns of* E. cava* extract (a) and its ethyl acetate fraction (b). The peaks of eckol and dieckol were identified by the comparison of the retention times with those of standard compounds. The chemical structures of eckol and dieckol are shown in (c) and (d).

**Figure 5:**
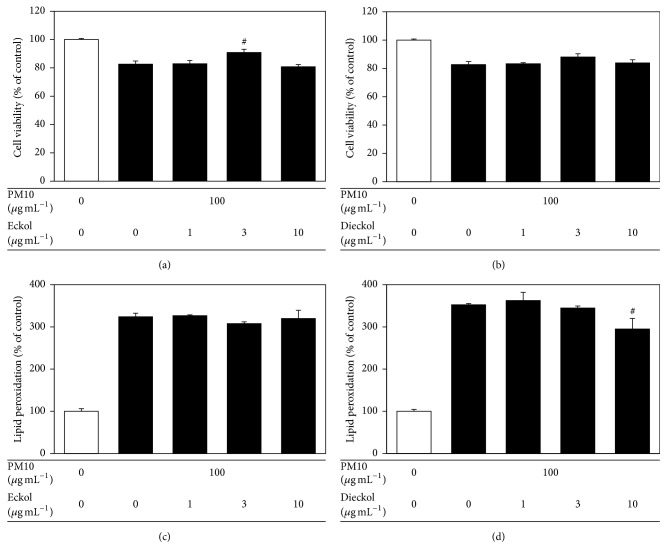
The effects of eckol and dieckol on viability of and lipid peroxidation in HaCaT keratinocytes exposed to PM10. The cells were exposed to PM10 (100 *μ*g mL^−1^) for 48 h in the absence and presence of eckol or dieckol at different concentrations (1–10 *μ*g mL^−1^). The cell viability (a, b) and cellular lipid peroxidation (c, d) were determined. The data are presented as a percentage of the control values (mean ± SE, *n* = 3). ^#^*p* < 0.05 versus PM10 control.

*E. cava* contains various polyphenolic compounds, including eckol and dieckol [17]. As shown in Figures 4(a) and 4(b),* E. cava* extract contained eckol and dieckol; these compounds were enriched in the ethyl acetate fraction. When tested in HaCaT cells exposed to PM10, eckol rescued cell viability at 3 *μ*g mL^−1^ and dieckol attenuated lipid peroxidation at 10 *μ*g mL^−1^ (Figure 5).

**Figure 6:**
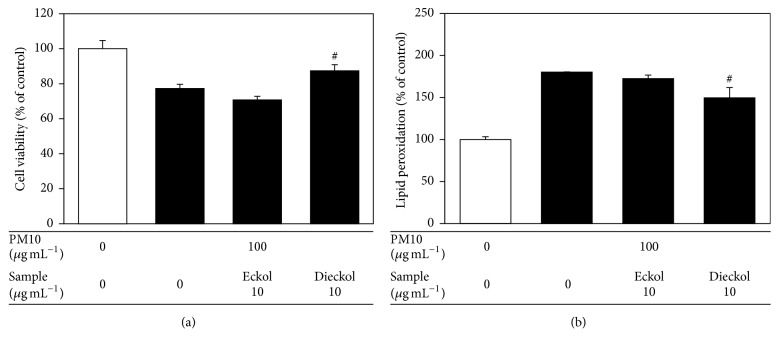
The effects of eckol and dieckol on viability of and lipid peroxidation in human epidermal keratinocytes exposed to PM10. The cells were exposed to PM10 (100 *μ*g mL^−1^) for 48 h in the absence and presence of eckol or dieckol (10 *μ*g mL^−1^). The cell viability (a) and cellular lipid peroxidation (b) were determined. The data are presented as the percentage of the control values (mean ± SE, *n* = 3). ^#^*p* < 0.05 versus PM10 control.

**Figure 7:**
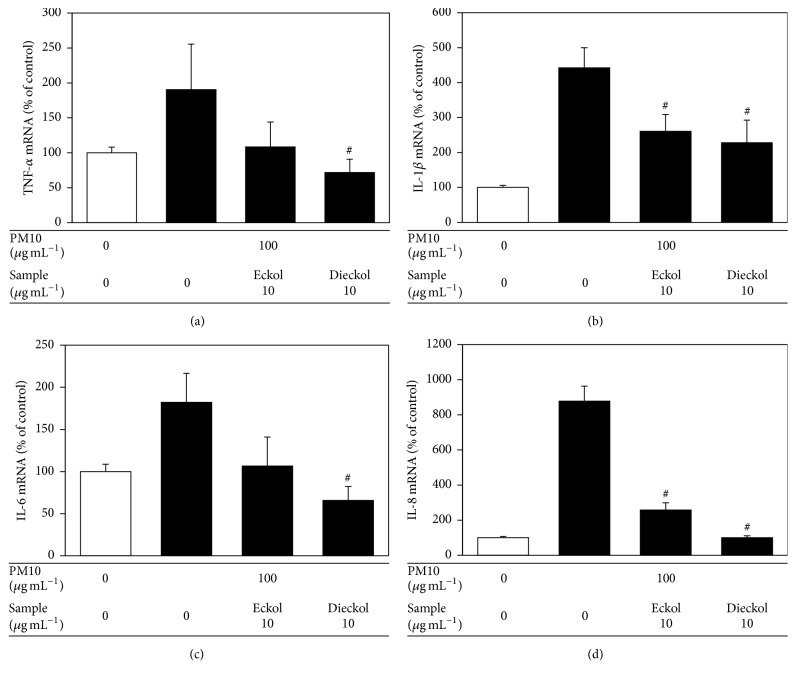
The effects of eckol and dieckol on the expression of inflammatory cytokines in human epidermal keratinocytes exposed to PM10. The cells were exposed to PM10 (100 *μ*g mL^−1^) for 24 h in the absence and presence of eckol or dieckol (10 *μ*g mL^−1^). The mRNA levels of TNF-*α* (a), IL-1*β* (b), IL-6 (c), and IL-8 (d) were analyzed by qRT-PCR and normalized to those of GAPDH, a housekeeping gene. The data are presented as the percentage of the control value (mean ± SE, *n* = 3). ^#^*p* < 0.05 versus PM10 control.

**Figure 8:**
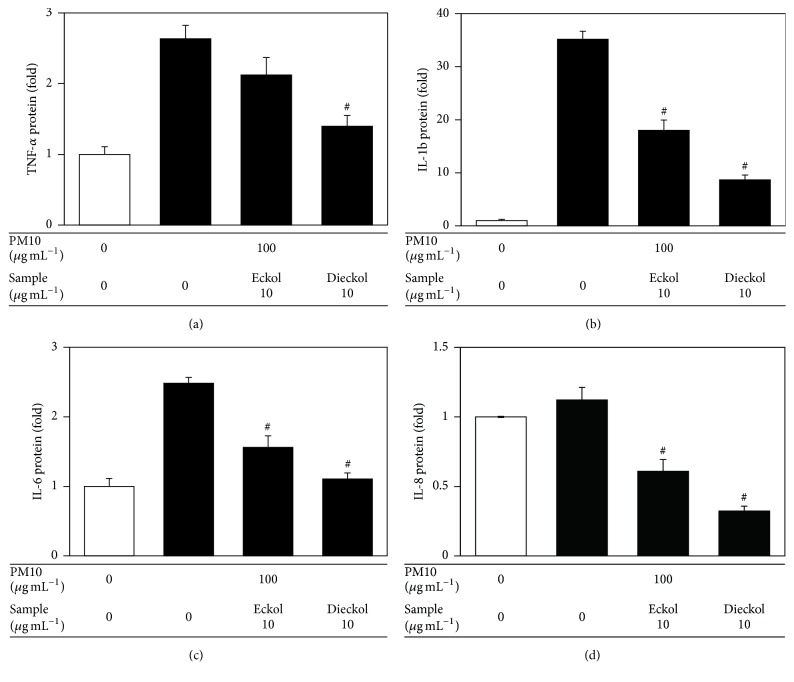
The effects of eckol and dieckol on the expression of inflammatory cytokines in human epidermal keratinocytes exposed to PM10. The cells were exposed to PM10 (100 *μ*g mL^−1^) for 48 h in the absence and presence of eckol or dieckol (10 *μ*g mL^−1^). The protein levels of TNF-*α*, IL-1*β*, IL-6, and IL-8 in the culture medium were analyzed by ELISA and normalized to the total protein content of the cells. Data are presented as fold changes compared to the control value (mean ± SE, *n* = 3). ^#^*p* < 0.05 versus PM10 control.

Human epidermal keratinocytes were also used to examine the antioxidant and anti-inflammatory effects of eckol and dieckol. Dieckol rescued cell viability and attenuated cellular lipid peroxidation in human epidermal keratinocytes exposed to PM10 (Figure 6). In the PM10-exposed human epidermal keratinocytes, dieckol attenuated the expression of inflammatory cytokines such as TNF-*α*, IL-1*β*, IL-6, and IL-8 at the mRNA and protein levels, more effectively than eckol (Figures 7 and 8).

## 4. Discussion

*E. cava* is a brown alga (Lessoniaceae) found in the coastal area of Korea and Japan and has been used as food and traditional medicine [18]. It is a rich source of phlorotannins and fucoidans that possesses a wide range of biological activities [17]. Phlorotannins are produced by the polymerization of phloroglucinol and are found only in marine brown algae.* E. cava* contains various phlorotannins, such as eckol, dieckol, 6,6′-bieckol, eckstolonol, and triphlorethol-A [19]. The biological effects of* E. cava* identified so far include antioxidant, anti-inflammatory, antibacterial, antidiabetic, and anticancer activities [20–25].

The present study demonstrated that the antioxidant effects of* E. cava* extract mitigated cellular lipid peroxidation in HaCaT keratinocytes exposed to PM10. In addition, one of its constituent compounds, dieckol, attenuated cellular lipid peroxidation and the expression of inflammatory cytokines in human epidermal keratinocytes exposed to PM10. Although the antioxidant and anti-inflammatory effects of* E. cava* extract have been reported in other experimental models, the present study was the first to describe the antioxidant and anti-inflammatory behavior in keratinocytes exposed to PM10.

The* E. cava* extract was found to attenuate PM10-induced cellular lipid peroxidation in a dose-dependent manner. Of the solvent fractions of* E. cava* extract, the ethyl acetate fraction attenuated the cellular lipid peroxidation most effectively. Water n-butyl alcohol fractions exhibited cytotoxicity. Thus, purification steps are necessary to improve the activity and safety profiles of* E. cava* extracts.

Eckol and dieckol are known polyphenolic constituents of* E. cava. *Dieckol is a dimeric form of eckol, and it is of interest to observe the different effects of these two compounds in cells. Compared with eckol, dieckol was more active against the PM10-induced cellular lipid peroxidation in both HaCaT and human epidermal keratinocytes. In addition, dieckol inhibited the expression of inflammatory cytokines in human epidermal keratinocytes, more strongly than eckol did.

The composition of airborne PM10 varies depending on the locations and seasons. PM10 usually contains miscellaneous toxic compounds, including transition metals, endotoxins, and ultrafine components, and induces oxidative stress and inflammation in various organs [26, 27]. It stimulates cellular reactive oxygen (ROS) production [28–30] and the expression of inflammatory cytokines such as TNF-*α* and IL-1*β* [31, 32]. In the present study, it was confirmed that PM10 increased cellular lipid peroxidation and induced the gene expression of the inflammatory cytokines TNF-*α*, IL-1*β*, IL-6, and IL-8. In addition, the PM10-induced lipid peroxidation and cytokine expression were attenuated by dieckol and eckol in human epidermal keratinocytes.

Previous studies have used cell culture models and reconstructed human epidermis models to investigate the harmful effects of airborne pollution on the skin [33–35]. The keratinocytes culture model used in the present study was useful in identifying potential antioxidants which might protect the skin exposed to air pollution. Further studies are needed to validate its antioxidative and anti-inflammatory effects against PM10* in vivo*.

In conclusion, this study suggested that the polyphenolic constituents of* E. cava,* such as dieckol, attenuated the oxidative and inflammatory reactions in skin cells exposed to airborne particulate matter.

## Figures and Tables

**Table 1:** Total phenol contents (TPC) of marine plants extracts used in this study.

Marine plant name	Catalogue number	Parts used	TPC^*∗*^ (*μ*g GAE mg^−1^ extract)
*Acrosorium yendoi* Yamada	JBRI-20419	Whole plants	42.70
*Agarum cribrosum* Bory de Saint-Vincent	JBRI-20086	Leaves	19.31
*Amphiroa anceps* (Lamarck) Decaisne	JBRI-20373	Whole plants	8.16
*Asparagopsis taxiformis* (Delile) Trevisan	JBRI-20407	Whole plants	17.76
*Bonnemaisonia hamifera* Hariot	JBRI-10278	Leaves	7.18
*Callophyllis japonica* Okamura	JBRI-20365	Whole plants	10.35
*Callophyllis crispata* Okamura	JBRI-10353	Leaves	3.28
*Carpopeltis affinis* (Harvey) Okamura	JBRI-10376	Leaves	5.63
*Champia parvula* (C. Agardh) Harvey	JBRI-20408	Whole plants	7.70
*Chondracanthus tenellus* (Harvey) Hommersand	JBRI-10270	Leaves	18.79
*Chondria crassicaulis* Harvey	JBRI-10264	Leaves	3.74
*Chondrus ocellatus* Holmes	JBRI-10267	Leaves	5.81
*Cladophora wrightiana* Harvey	JBRI-20396	Whole plants	16.67
*Codium fragile* (Suringar) Hariot	JBRI-20092	Leaves	1.95
*Colpomenia sinuosa* (Mertens ex Roth) Derbes et Solier	JBRI-10355	Leaves	2.41
*Costaria costata* (C. Agardh) Saunders	JBRI-20020	Leaves	2.76
*Desmarestia tabacoides* Okamura	JBRI-20087	Leaves	7.24
*Dictyopteris dichotoma* (Hudson) Lamouroux	JBRI-20096	Leaves	6.95
*Dictyota okamurae *(Dawson) Hötning, Schnetter, et Prud'homme van Reine	JBRI-20435	Whole plants	12.07
*Distromium decumbens *Okamura	JBRI-20161	Leaves	52.36
*Ecklonia cava* Kjellman	JBRI-10282	Leaves	89.43
*Eisenia bicyclis* (Kjellman) Setchell	JBRI-20022	Leaves	39.14
*Galaxaura falcata* Kjellman	JBRI-20367	Whole plants	6.72
*Gelidium amansii* (Lam.) Lamouroux	JBRI-10244	Leaves	7.30
*Gloiopeltis complanata* (Harvey) Yamada	JBRI-10527	Leaves	33.10
*Gracilaria verrucosa* (Hudson) Papenfuss	JBRI-10698	Leaves	5.23
*Grateloupia crispata *(Okamura) Lee	JBRI-20366	Whole plants	5.29
*Grateloupia elliptica* Holmes	JBRI-10263	Leaves	3.22
*Hydroclathrus clathratus* (C. Agardh) Howe	JBRI-10366	Leaves	0.29
*Hypnea charoides *Lamouroux	JBRI-20378	Whole plants	9.31
*Ishige okamurae* Yendo	JBRI-10276	Leaves	29.20
*Laurencia intermedia* Lamouroux	JBRI-10351	Leaves	3.68
*Leathesia difformis* (Linnaeus) Areschoug	JBRI-20346	Whole plants	4.20
*Lomentaria hakodatensis* Yendo	JBRI-10266	Leaves	4.028
*Martensia denticulata* Harvey	JBRI-20221	Leaves	36.72
*Myagropsis myagroides* (Martens ex Turner) Fensholt	JBRI-20370	Whole plants	2.36
*Myelophycus simplex* (Harvey) Papenfuss	JBRI-10262	Leaves	2.30
*Pachydictyon coriaceum* (Holmes) Okamura	JBRI-20110	Leaves	5.58
*Padina arborescens* Holmes	JBRI-10172	Leaves	5.00
*Petalonia binghamiae* (J. Agardh) Vinogradova	JBRI-10173	Leaves	8.28
*Phyllospadix iwatensis* Makino	JBRI-10700	Leaves	23.79
*Plocamium telfairiae* (Harvey) Harvey	JBRI-20039	Leaves	11.67
*Prionitis cornea* (Okamura) Dawson	JBRI-10272	Leaves	5.12
*Pterocladia capillacea* (Gmelin) Bornet	JBRI-20027	Leaves	6.38
*Sargassum fusiformis* (Harvey) Okamura	JBRI-10495	Leaves	2.93
*Scytosiphon gracilis* Kogame	JBRI-10517	Leaves	3.10
*Ulothrix flacca* (Dillwyn) Thuret	JBRI-20359	Whole plants	1.38
*Ulva fasciata* Delile	JBRI-20025	Leaves	4.37
*Undaria pinnatifida* (Harvey) Suringar	JBRI-20051	Leaves	1.27
*Zostera marina* L.	JBRI-20093	Leaves	4.43

## References

[1] Sacks J. D., Stanek L. W., Luben T. J. (2011). Particulate matter-induced health effects: who is susceptible?. *Environmental Health Perspectives*.

[2] Anderson J. O., Thundiyil J. G., Stolbach A. (2012). Clearing the air: a review of the effects of particulate matter air pollution on human health. *Journal of Medical Toxicology*.

[3] Donaldson K., Stone V., Clouter A., Renwick L., MacNee W. (2001). Ultrafine particles. *Occupational and Environmental Medicine*.

[4] Li X. Y., Gilmour P. S., Donaldson K., MacNee W. (1996). Free radical activity and pro-inflammatory effect of particulate air pollution (PM10) in vivo and in vitro. *Thorax*.

[5] Guo L., Zhu N., Guo Z. (2012). Particulate matter (PM_10_) exposure induces endothelial dysfunction and inflammation in rat brain. *Journal of Hazardous Materials*.

[6] Hwang S. H., Choi Y.-H., Paik H. J., RyangWee W., KumKim M., Kim D. H. (2016). Potential importance of ozone in the association between outdoor air pollution and dry eye disease in South Korea. *JAMA Ophthalmology*.

[7] Kim K. E., Cho D., Park H. J. (2016). Air pollution and skin diseases: Adverse effects of airborne particulate matter on various skin diseases. *Life Sciences*.

[8] Vierkötter A., Schikowski T., Ranft U. (2010). Airborne particle exposure and extrinsic skin aging. *Journal of Investigative Dermatology*.

[9] Villarreal-Calderon R., Reed W., Palacios-Moreno J. (2012). Urban air pollution produces up-regulation of myocardial inflammatory genes and dark chocolate provides cardioprotection. *Experimental and Toxicologic Pathology*.

[10] Saputri R. K., Setiawan B., Nugrahenny D., Kania N., Sri Wahyuni E., Widodo M. A. (2014). The effects of *Eucheuma cottonii* on alveolar macrophages and malondialdehyde levels in bronchoalveolar lavage fluid in chronically particulate matter 10 coal dust-exposed rats. *Iranian Journal of Basic Medical Sciences*.

[11] Park S., Seok J. K., Kwak J. Y., Suh H.-J., Kim Y. M., Boo Y. C. (2016). Anti-Inflammatory Effects of Pomegranate Peel Extract in THP-1 Cells Exposed to Particulate Matter PM10. *Evidence-Based Complementary and Alternative Medicine*.

[12] Kiuru P., Valeria D’Auria M., Muller C. D., Tammela P., Vuorela H., Yli-Kauhaluoma J. (2014). Exploring marine resources for bioactive compounds. *Planta Medica*.

[13] Cha S.-H., Ko S.-C., Kim D., Jeon Y.-J. (2011). Screening of marine algae for potential tyrosinase inhibitor: those inhibitors reduced tyrosinase activity and melanin synthesis in zebrafish.. *The Journal of Dermatology*.

[14] Heo S.-J., Ko S.-C., Kang S.-M. (2010). Inhibitory effect of diphlorethohydroxycarmalol on melanogenesis and its protective effect against UV-B radiation-induced cell damage. *Food and Chemical Toxicology*.

[15] Kwak J. Y., Seok J. K., Suh H.-J. (2016). Antimelanogenic effects of luteolin 7-sulfate isolated from Phyllospadix iwatensis Makino. *British Journal of Dermatology*.

[16] Uchiyama M., Mihara M. (1978). Determination of malonaldehyde precursor in tissues by thiobarbituric acid test. *Analytical Biochemistry*.

[17] Wijesinghe W. A. J. P., Jeon Y.-J. (2012). Exploiting biological activities of brown seaweed Ecklonia cava for potential industrial applications: A review. *International Journal of Food Sciences and Nutrition*.

[18] Kim M.-M., Ta Q. V., Mendis E. (2006). Phlorotannins in Ecklonia cava extract inhibit matrix metalloproteinase activity. *Life Sciences*.

[19] Li Y., Qian Z.-J., Ryu B., Lee S.-H., Kim M.-M., Kim S.-K. (2009). Chemical components and its antioxidant properties in vitro: An edible marine brown alga, Ecklonia cava. *Bioorganic & Medicinal Chemistry*.

[20] Lee H., Kang C., Jung E.-S., Kim J.-S., Kim E. (2011). Antimetastatic activity of polyphenol-rich extract of Ecklonia cava through the inhibition of the Akt pathway in A549 human lung cancer cells. *Food Chemistry*.

[21] Choi J.-G., Kang O.-H., Brice O.-O. (2010). Antibacterial activity of *Ecklonia cava* against methicillin-resistant *Staphylococcus aureus* and *Salmonella* spp. *Foodborne Pathogens and Disease*.

[22] Kang C., Jin Y. B., Lee H. (2010). Brown alga Ecklonia cava attenuates type 1 diabetes by activating AMPK and Akt signaling pathways. *Food and Chemical Toxicology*.

[23] Shin H.-C., Hwang H. J., Kang K. J., Lee B. H. (2006). An antioxidative and antiinflammatory agent for potential treatment of osteoarthritis from Ecklonia cava. *Archives of Pharmacal Research*.

[24] Kang S.-M., Cha S.-H., Ko J.-Y. (2012). Neuroprotective effects of phlorotannins isolated from a brown alga, Ecklonia cava, against H_2_O_2_-induced oxidative stress in murine hippocampal HT22 cells. *Environmental Toxicology and Pharmacology*.

[25] Heo S.-J., Ko S.-C., Cha S.-H. (2009). Effect of phlorotannins isolated from Ecklonia cava on melanogenesis and their protective effect against photo-oxidative stress induced by UV-B radiation. *Toxicology in Vitro*.

[26] Donaldson K., Stone V. (2003). Current hypotheses on the mechanisms of toxicity of ultrafine particles. *Ann Ist Super Sanita*.

[27] Ishii H., Fujii T., Hogg J. C. (2004). Contribution of IL-1*β* and TNF-*α* to the initiation of the peripheral lung response to atmospheric particulates (PM_10_). *American Journal of Physiology-Lung Cellular and Molecular Physiology*.

[28] Cho D.-Y., Le W., Bravo D. T. (2014). Air pollutants cause release of hydrogen peroxide and interleukin-8 in a human primary nasal tissue culture model. *International Forum of Allergy & Rhinology*.

[29] Bedard K., Krause K. (2007). The NOX family of ROS-generating NADPH oxidases: physiology and pathophysiology. *Physiological Reviews*.

[30] Lassègue B., Griendling K. K. (2010). NADPH oxidases: functions and pathologies in the vasculature. *Arteriosclerosis, Thrombosis, and Vascular Biology*.

[31] Bengalli R., Molteni E., Longhin E., Refsnes M., Camatini M., Gualtieri M. (2013). Release of IL-1*β* triggered by milan Summer PM_10_: molecular pathways involved in the cytokine release. *BioMed Research International*.

[32] Fujii T., Hayashi S., Hogg J. C. (2002). Interaction of alveolar macrophages and airway epithelial cells following exposure to particulate matter produces mediators that stimulate the bone marrow. *American Journal of Respiratory Cell and Molecular Biology*.

[33] Lecas S., Boursier E., Fitoussi R. (2016). In vitro model adapted to the study of skin ageing induced by air pollution. *Toxicology Letters*.

[34] Soeur J., Belaïdi J.-P., Chollet C. (2017). Photo-pollution stress in skin: Traces of pollutants (PAH and particulate matter) impair redox homeostasis in keratinocytes exposed to UVA1. *Journal of Dermatological Science*.

[35] Lin Z.-C., Lee C.-W., Tsai M.-H. (2016). Eupafolin nanoparticles protect HaCaT keratinocytes from particulate matter-induced inflammation and oxidative stress. *International Journal of Nanomedicine*.

